# Ophthalmology emergency department visits in a Brazilian tertiary hospital over the last 11 years: data analysis

**DOI:** 10.5935/0004-2749.20230067

**Published:** 2023

**Authors:** Lucas Zago Ribeiro, Luis Filipe Nakayama, Vinicius Campos Bergamo, Caio Vinicius Saito Regatieri

**Affiliations:** 1 Department of Ophthalmology, Escola Paulista de Medicina, Universidade Federal de São Paulo, São Paulo, SP, Brazil.

**Keywords:** Emergency service, hospital, Epidemiology, Eye injuries, Eye diseases, Serviço hospitalar de emergência, Epidemiologia, Traumatismos oculares, Oftalmopatias

## Abstract

**Purpose:**

This study aimed to describe the visits profile to Hospital São Paulo’s ophthalmology emergency department, a 24-hour public open-access tertiary-care service in São Paulo, Brazil, that belongs to Federal University of São Paulo, over the last 11 years.

**Methods:**

A cross-sectional retrospective study was conducted, including all patients (n=634,726) admitted to the ophthalmology emergency department of Hospital São Paulo between January 2009 and December 2019.

**Results:**

From 2009 to 2019, the number of patients’ presentations increased to 39.2%, with considerable visits variation across the period. The median age was 38 ± 20.4 years. Males represented 53.3%, and single-visit patients represented 53.1%. A total of 79.5% of patients’ presentations occurred from 7 am to 5 pm, and 80.8% of patients’ presentations occurred during regular weekdays. The most frequent diagnoses were conjunctivitis, blepharitis, keratitis, hordeolum/chalazion, and corneal foreign body.

**Conclusions:**

Over the study period, presentations significantly increased in number, with nonurgent visits predominance, and a low number of single-visit patients. Our results demonstrate the ophthalmic visits profile and can lead to changes in the public health system to improve the quality of care and ophthalmology emergency access in São Paulo city.

## Abstract (pt)

**Objetivos:**

O objetivo do estudo é avaliar o perfil das visitas ao Pronto-Socorro de Oftalmologia (PS) do Hospital São Paulo, serviço público de atendimento terciário aberto 24 horas em São Paulo - Brasil, pertencente à Universidade Federal de São Paulo, nos últimos 11 anos.

**Métodos:**

Foi realizado um estudo transversal retrospectivo, com base em todos os pacientes (n=634.726) admitidos no pronto-socorro de oftalmologia do Hospital São Paulo entre janeiro de 2009 e dezembro de 2019.

**Resultados:**

De 2009 a 2019, houve um aumento no influxo de 39,2% com importante variação nos atendimentos ao longo dos anos, a mediana de idade foi de 38 ± 20,4 anos, o sexo masculino representou 53,3% e os pacientes únicos representaram 53,1%. Verificou-se que 79,5% das visitas ocorreram das 7h às 17h e 80,8% nos dias da semana. Os diagnósticos mais frequentes foram conjuntivite aguda seguida de blefarite, ceratite, hordéolo / calázio e corpo estranho corneano.

**Conclusão:**

Ao longo do período de análise do estudo, houve importante aumento nas apresentações, com predominância de atendimentos não urgentes e baixa proporção de pacientes com uma única visita. Nossos resultados evidenciam o perfil das consultas oftalmológicas, podendo gerar mudanças no sistema público de saúde visando a melhoria da qualidade do atendimento e acesso às emergências oftalmológicas na cidade de São Paulo.

## INTRODUCTION

Emergency departments (EDs) are an essential part of patient care, with the unique capability to provide 24-hour full-range immediate medical services^(1)^. Conditions that require urgent ocular care, such as ocular trauma, infections, retinal detachment, and uveitis, are associated with a high risk of visual impairment if they do not receive appropriate treatment^(2)^. Despite representing a small body surface, the eyes are the third most frequent organ, after hands and feet, affected by injuries^(3)^. Besides, vision is an essential overall health quality aspect, and vision loss is a significant risk factor for functional decline^(4)^.

However, crowding ophthalmology EDs is a real situation in most countries^(1,3,5)^, leading to delayed and low-quality care for real urgent cases. Nonurgent visits, such as those for glass prescription, dry eye syndrome, blepharitis, and chalazion, have been reported between 8% and 62% of total patients’ visits^(6)^, especially at self-referral services.

The high number of nonurgent visits to EDs is an issue described in previous studies, and it is probably a significant aspect of crowding in waiting rooms and delay in medical care. In Brazil, we have another important factor, as the majority of the population depends only on our public health system (SUS-Sistema Único de Saúde) to access health care, which is universally accessible and free. SUS is divided into three care complexity levels (primary, secondary, and tertiary care) that should work as an integrated network to organize patient access from primary care to the other levels^(7)^.

Many patients seek ED attendance for nonurgent complaints, probably because of lacking information and facing difficulties in accessing ophthalmological assistance in primary care^(6)^. For years, the challenges in access to care could make originally nonurgent cases arrive at the emergency room with an advanced disease phase having a poor prognosis and demanding an urgent intervention, such as in cases of glaucoma and diabetic retinopathy.

There are few studies on ophthalmology ED visits profiles in Brazilian hospitals, especially assessing trends from the last 5 years. Only a few studies in the world analyzed abundant data from ophthalmology visits.The *Universidade Federal de São Paulo* (UNIFESP) ophthalmology ED is linked to Hospital São Paulo, a tertiary-care 24-hour public open-access hospital located in São Paulo, which belongs to UNIFESP. Despite high visits volume, there is no ophthalmological triage system in the hospital. In addition to healthcare purposes, it can offer education for residents in training.

This study aimed to evaluate the visits profile to UNIFESP ED over the last 10 years, evaluating the causes for the change in inflow and possible proposals to improve the service flow.

## METHODS

A cross-sectional retrospective study was conducted based on data analysis from all patients admitted to the ophthalmology ED of Hospital São Paulo from January 2009 to December 2019.

This study was approved by the Institutional Ethics Committee of UNIFESP and followed Helsinki principles.

Hospital São Paulo is a state-funded, free 24/7 emergency hospital in São Paulo, Brazil, with an assistance area in the city’s South Zone, covering a 5-million population. The permanent staff comprises ophthalmology residents (four during regular diurnal and 2 nocturnal weekdays and weekends full-time) and two ophthalmologists.

The data were collected from the electronic medical records available in the hospital database. The following data that coordinates from ED medical charts were retrieved by the hospital information technology specialists: patient-internal registration code, date and hour, age at the visit, sex, informed zip code, and ICD-10 (International Classification of Diseases-10), as informed by the physician. ICD-10 chapter 7 (“Diseases of the eye and adnexa”, codes H00-H59) and chapter 19 (“Injury, poisoning and certain other consequences of external causes”, codes S00-T88) were used.

Retrieved data were compiled in an anonymized spreadsheet for subsequent statistical analysis.

Initially, all patients were considered for statistical analysis. In postanalysis, we excluded patients without identifiable diagnoses and completed medical care records.

Among different data, epidemiologic parameters, medical diagnosis, number of visits according to day hour, day of the week, month and year, and number of visits according to medical staff were analyzed.

## RESULTS

**Figure 1:**
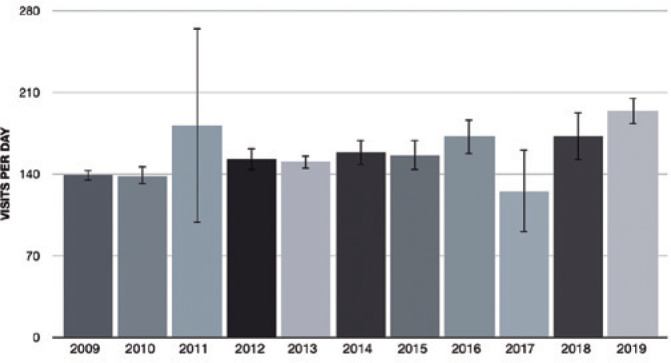
Visits per day in ophthalmology emergency department from January 2009 to December 2019 in São Paulo - Brazil (Visits per day and standard deviation error bars).

During the 11 years of the study, there were 634,726 visits to Hospital São Paulo ophthalmology ED, with a mean of 57,702 ± 7,390.5 per year (± standard deviation), going from 50,729 in 2009 to 70,623 visits in 2019, representing an increase at the inflow of 19,854 (39.2%) (Table 1 and Figure 1).

**Table 1:** Mean visits per day during each quarter of 2009 to 2019

Years	Q1	Q2	Q3	Q4	TOTAL	SD	N
**2009**	137.6	140.4	136.3	141.5	**134.0**	**4.5**	50,729
**2010**	144.0	132.8	138.1	139.9	**138.6**	**7.7**	50,586
**2011**	252.8	185.6	136.7	151.0	**181.3**	**83.8**	66,192
**2012**	154.4	149.2	154.4	153.7	**152.4**	**9.3**	55,789
**2013**	149.1	147.8	150.8	153.0	**150.2**	**5.7**	54,819
**2014**	162.4	151.7	160.0	159.5	**158.3**	**10.5**	57,795
**2015**	159.1	153.5	141.6	169.8	**156.0**	**12.8**	56,928
**2016**	168.4	161.5	183.9	175.6	**171.8**	**15.1**	62,880
**2017**	172.2	84.0	109.4	136.1	**125.2**	**35.5**	45,711
**2018**	158.8	163.6	167.9	196.4	**171.7**	**20.5**	62,674
**2019**	184.7	186.6	200.5	202.0	**193.5**	**11.4**	70,623
**TOTAL**							634,726

Q1= First quarter; Q2= Second quarter; Q3= Third quarter; Q4= Fourth quarter; SD= Standard deviation; N= total visits per year.

The analysis of the number of visits per day showed a mean of 158.1 ± 34.3 visits per day. The month that showed the highest inflow was March 2011, with 410.9 visits per day, and the lowest inflow was in April 2017, with 65.5 visits per day. While evaluating the inflow and excluding 2011 (181.4 ± 83.8 visits per day) and 2017 (125.2 ± 20.5) from the analysis, as they were out of pattern compared to the year before and after each one, the highest inflow was during the fourth quarter, with 165.7 visits per day, and the lowest during the second quarter, with 154.1 visits per day (Table 1 and Figure 1).

The percentage of single-visit patients through the years remained between 64.7% in 2009 (lowest rate) and 72.2% in 2019 (highest percentage). When evaluating a period of 11 years, single-visit patients represented 53.1% of the total visits, which means that 336,704 patients single visited the ED during this period.

The median patient age was 38 ± 20.4 years (range 0-101), where patients under 5 years represented 4.8%, and patients over 65 represented 12.4%. The age profile did not show a significant change over the years, with the lowest mean of 39.3 ± 20.5 years in 2009 and the highest mean of 41.2 ± 20.0 in 2019. Male patients represented 54.3% of the total proportion of visits (Table 2).

**Table 2:** Demographic profile of patients examined in ophthalmology emergency department from January 2009 to December 2019

	2009-2019		
	**N**	**%**	
Male	344,783	54.3	
Female	289,943	45.7	
Age (years)	**n**	**%**	**SD**
0-5	30,412	4.8	380.2
6-15	39,200	6.3	503.0
16-30	162,166	25.7	25556.6
31-45	157,106	24.6	2141.8
46-65	168,007	26.3	2411.7
>65	78,531	12.3	899.9

SD= Standard deviation.

The visits showed substantial variation when comparing regular weekdays and weekends, a variation that was a common pattern over the years. Regular weekday visits represented 80.8% ± 1.4% while analyzing the entire study period, with the lowest percentage of 79.7% in 2019 and the highest percentage of 82.6% of 2011. Visits on Mondays corresponded to 18.2% ± 0.6%, and those on Sundays corresponded to only 7.8% ± 0.5%. Inflow rates tended to progressively reduce from Monday to Friday (Table 3).

**Table 3:** Timing of visits to ophthalmology emergency department from January 2009 to December 2019

Timing of visits	2009-2019		SD
	Visits per day	% of total	
Monday	201.7	18.2	26.7
Tuesday	184.4	16.7	22.3
Wednesday	179.1	16.2	22.0
Thursday	170.4	15.4	23.9
Friday	158.4	14.3	19.6
Saturday	126.3	11.4	15.2
Sunday	85.8	7.8	14.2
	**Visits per day**	**% of total**	**SD**
12 am-4:59 am	2.7	1.7	1.1
5 am-6:59 am	4.3	2.7	3.2
7 am-9:59 am	41.8	26.5	4.8
10 am-12:59 pm	45.1	28.5	5.8
1 pm-4:59 pm	38.6	24.5	5.1
5 pm-7:59 pm	14.1	8.9	2.6
8 pm-11:59 pm	11.5	7.3	2.9

SD= Standard deviation.

The analysis per day period showed that 79.4% of visits occurred from 7 am to 5 pm. The inflow significantly increased between 8 pm and midnight, being 5.8% in 2009 (mean of 8.2 visits) and 9% in 2019 (mean of 17.4), and also between midnight and 5 am, being 1.1% (mean of 1.5 visits) in 2009 and 2.6% (mean of 4.9 visits) in 2019 (Table 3).

**Figure 2:**
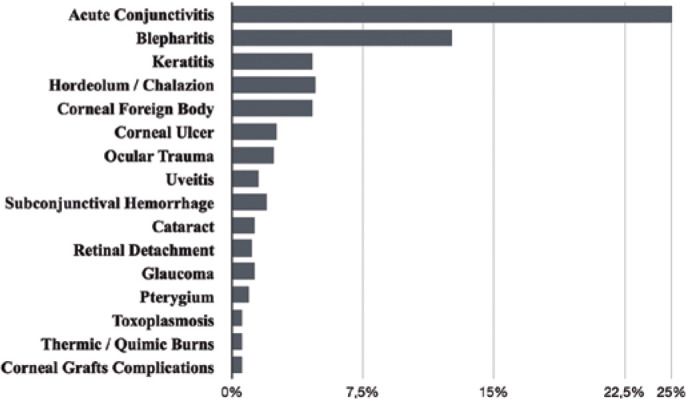
Leading diagnoses in ophthalmology emergency department from January 2015 to December 2019 in São Paulo - Brazil.

The most commonly physician-reported ICD-10 diagnoses were acute conjunctivitis, blepharitis, keratitis, corneal foreign body, subconjunctival hemorrhage, and ocular trauma. ICD-10 data between 2009 and 2014 were not considered for analysis due to a large amount of incomplete data. The analysis between 2015 and 2019, excluding files with missing data (21%), showed that acute conjunctivitis represented 34% (H10), blepharitis 1 represented 6.9% (H01.0), keratitis represented 7% (H16.1; H16.3; H16.8), hordeolum/chalazion represented 6.4% (H00), corneal foreign body represented 6.2% (T15.0), corneal ulcer represented 3.5% (H16.0), ocular trauma represented 3.2% (S05), and subconjunctival hemorrhage represented 2.8% (H11.3) (Figure 2).

## DISCUSSION

Emergency consultation is essential to properly manage urgent health problems, such as ocular trauma, uveitis, infections, and retina detachments^(2)^. However, it provides quick access to ophthalmological evaluation, specifically in the Brazilian health system. Since adequate ophthalmological covering and populational orientation are lacking, crowded EDs are a real issue in Brazil. The number of public ophthalmology EDs in São Paulo has decreased or limited its access over the last years, which could also explain the increased crowding of our service.

The increase of 19,854 (+39.2%) visits from 2009 (50,729) to 2019 (70,623) is a significant change in inflow, representing more visits than many previous studies showed per year^(7-10)^. This can be explained by the improvement of hospital accessibility and public transport around our ED, such as with the construction of a subway station close to the hospital in 2018. Other explanations include the closure of some public open-access ophthalmology EDs in São Paulo and the reduced access to private health systems in the Brazilian population^(11)^. Our assistance area covers a 5-million population area in the South Zone of São Paulo but our daily experience shows that many patients come from other zones or even from other federative units in Brazil, and a future analysis based on patient origin is required for a better understanding of the underlying reasons of this phenomenon.

São Paulo experienced an acute conjunctivitis epidemic, which corresponds to the abruptly increased inflow during the first semester in 2011^(12)^. UNIFESP ED was not completely open for visits during the second quarter of 2017, which explains the decreased inflow.

Data from previous studies on Brazilian ophthalmology EDs showed 1,224 visits in 3 months in 2000 (13.6 per day) at service in Sergipe, Brazil^(9)^, 581 visits per week (83 per day) during 2006 at a tertiary hospital in São Paulo, Brazil^(7)^, 8,346 visits in 5 months of 2005 (55.6 per day) at a tertiary hospital in Belo Horizonte, Brazil^(8)^, and 8,689 visits during 2009 (32.8 per day) at a tertiary hospital in Goiânia, Brazil^(10)^. We could not find newly published studies that evaluated Brazilian ophthalmology EDs from the last 5 years or evaluated such a high number of visits (70,623 visits in 2019 with a mean of 193.5 visits per day).

The comparison with previous Brazilian studies shows different situations in cities in Brazil and the lack of recent studies for comparison. We believe that the increase in volume and a high number of nonurgent visits are an issue in most Brazilian public ophthalmological services.

The increase of 39.2% found in our analysis is higher compared to previous studies from other countries. A previous study compared the change in eye-related trends to their ED in Beirut, Lebanon, from 1997 to 2012, finding a less significant increase in the inflow of 39,158 to 46,363 (+ 18%) during a 15-years period^(13)^. Another similar study found an increase in the inflow of 11% from 2001 to 2014 based on data analysis from more than 11 million visits in the mentioned period in the United States^(14)^. Comparing profiles by seasonal distribution, 27,120 visits were evaluated in 2013 at Turkey ophthalmology ED^(1)^.

The most common diagnoses comprised acute conjunctivitis, blepharitis, keratitis, corneal foreign body, and hordeolum/chalazion, which is a similar profile compared to previous reports^(7,9,15-17)^. In previous reports, corneal foreign bodies appear as the most common diagnosis^(9,15-17)^. Another study found conjunctivitis, followed by a corneal foreign body, as the most common diagnosis at a tertiary hospital in São Paulo in 2006^(7)^. Nonurgent diseases, such as hordeolum and blepharitis, represent more than 23% of *Hospital São Paulo* ED cases. A deeper analysis of each file during a shorter period could help better differentiate between urgent and nonurgent cases and their social-demographic profile and clinical evolution.

The ophthalmic coverage and access in our area have been decreasing over the years, and patients have tried to use the ED service as a triage service. However, this does not represent an appropriate counter-referral system that could accept those patients after visiting the ED. Urgent cases are mostly accepted by UNIFESP Ambulatory Eye Clinics, when possible.

The low proportion of single-visit patients of only 53.1% (336,704), considering the entire study period, could be explained by representations from outpatient care UNIFESP Ambulatory Eye Clinics and a low success rate to follow up after initial ED consultation as initial healthcare service, necessitating more than one ED consultation.

Provided the lack of a well-established triaging system in ophthalmology, it becomes even harder to manage the high volume of patients every day in ED. Rome Eye Scoring System for Urgency and Emergency (RESCUE) was proposed by Rossi in 2007 and tried to establish a tested and effective way to triage ophthalmology patients^(18,19)^. It could be a way to apply a triaging system to our service, possibly adapting it to our reality.

The reduction of access to 24-hour public ophthalmic emergency services in São Paulo, associated with the inappropriate use of emergency services with nonurgent conditions, resulted from low population’s understanding and difficulty accessing ophthalmological services at primary care. Besides creating new public ophthalmic services, their collaboration is required to distribute patient care better and not overload a few ones. It is also vital to optimize the referral system, which could reduce nonurgent visits overloading emergency services.

A previous study in Wilmer Eye Institute (Baltimore, USA) found that allowing same-day access to ambulatory ophthalmology clinics decreases costs to the healthcare system and volume to ED^(5)^. Overcrowded EDs result in decreased patient satisfaction and increased physicians’ burnout, which is even more expensive for the healthcare system, an important aspect for a public system like SUS in Brazil. Stagg et al. agreed that facilitating clinics access is potentially the most effective way to manage nonurgent cases out of emergency care^(14)^.

Our study has several limitations. Our data lack information on examination findings. Also, ICD-10 was provided by many different doctors and during different training stages, potentially resulting in misdiagnosis. The long period and a large amount of data included for analysis increase biases and possible errors in data that could not be checked. Despite the risk of bias, large data analysis, such as our study, can give a better understanding of the global change in our ED profile over the years, allowing appropriate adjustments to improve the quality of provided service.

In conclusion, overcrowded EDs are a real issue in Brazil, with a visits increase of 39.2% from 2009 to 2019 in UNIFESP ED. The hypothesis is that the reduction in emergency services in São Paulo city and inappropriate use of emergency services led to this problem. Solutions would comprise a triage system of urgent cases, remodeling the healthcare system to facilitate access to ambulatory clinics, and educational programs.
